# TRIM14 Promotes Noncanonical NF‐κB Activation by Modulating p100/p52 Stability via Selective Autophagy

**DOI:** 10.1002/advs.201901261

**Published:** 2019-11-11

**Authors:** Meixin Chen, Zhiyao Zhao, Qingcai Meng, Puping Liang, Zexiong Su, Yaoxing Wu, Junjiu Huang, Jun Cui

**Affiliations:** ^1^ State Key Laboratory of Oncology in South China MOE Key Laboratory of Gene Function and Regulation School of Life Sciences Sun Yat‐Sen University Guangzhou Guangdong 510006 China; ^2^ Department of Internal Medicine Guangzhou Institute of Pediatrics Guangzhou Women and Children's Medical Center Guangzhou Guangdong 510623 China

**Keywords:** inflammation, noncanonical NF‐κB signaling, p100/p52, selective autophagy, TRIM14

## Abstract

The noncanonical NF‐κB signaling pathway plays a critical role in a variety of biological functions including chronic inflammation and tumorigenesis. Activation of noncanonical NF‐κB signaling largely relies on the abundance as well as the processing of the NF‐κB family member p100/p52. Here, TRIM14 is identified as a novel positive regulator of the noncanonical NF‐κB signaling pathway. TRIM14 promotes noncanonical NF‐κB activation by targeting p100/p52 in vitro and in vivo. Furthermore, a mechanistic study shows that TRIM14 recruits deubiquitinase USP14 to cleave the K63‐linked ubiquitin chains of p100/p52 at multiple sites, thereby preventing p100/p52 from cargo receptor p62‐mediated autophagic degradation. TRIM14 deficiency in mice significantly impairs noncanonical NF‐κB‐mediated inflammatory responses as well as acute colitis and colitis‐associated colon cancer development. Taken together, these findings establish the TRIM14‐USP14 axis as a crucial checkpoint that controls noncanonical NF‐κB signaling and highlight the crosstalk between autophagy and innate immunity.

## Graphical abstract

TRIM14 is a novel positive regulator of the noncanonical NF‐κB signaling pathway. It recruits deubiquitinase USP14 to cleave the K63‐linked ubiquitin chains of p100/p52 at multiple sites, thereby preventing p100/p52 from cargo receptor p62‐mediated autophagic degradation. TRIM14 deficiency in mice significantly impairs noncanonical NF‐κB‐mediated inflammatory responses as well as acute colitis and colitis‐associated colon cancer development.

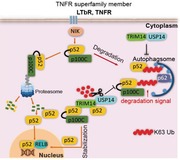

## 1 Introduction

The transcription factor NF‐κB family controls a variety of biological processes and plays a particular critical role throughout the immune system.^1^, ^2^, ^3^ In mammalians, the NF‐κB family consists of RelA (p65), RelB, c‐Rel, NF‐κB1 (p105), and NF‐κB2 (p100), which are activated by two major pathways: the canonical and the noncanonical NF‐κB pathways.^3^, ^4^ The activation of the canonical NF‐κB pathway depends on the proteasomal degradation of IκBα , leading to the activation of the p50, p65, and c‐Rel transcription factors.^5^ By contrast, the noncanonical NF‐κB pathway largely relies on the selective degradation of the protein precursor p100 (termed p100 processing), which yields the protein fragment termed p52 that acts as a transcription factor following nuclear translocation of p52‐RelB dimer.^3^, ^6^, ^7^

Under resting conditions, p100 harbors a carboxy‐terminal IκB‐homologous domain that serves as an IκB‐like inhibitor in the noncanonical NF‐κB pathway.^7^, ^8^ The stimulus‐induced NF‐κB‐inducing kinase (NIK) tightly integrates signals from a series of TNF receptor family members and activates the downstream kinase, IκB kinase‐α (IKKα), triggering p100 phosphorylation and processing.^3^, ^9^ Genetic ablation of murine NIK has revealed its importance in the noncanonical NF‐κB pathway.^10^ So far, several negative regulators of NIK have been identified. TRAF3 and TRAF2 could recruit cIAP1/2 to catalyze K48‐linked ubiquitination and subsequent degradation of NIK.^11^, ^12^ NLRP12 or OTUD7B stabilizes TRAF3 and subsequently accelerates NIK degradation.^13^, ^14^ Lastly, IKKα and TBK1 may induce NIK phosphorylation‐dependent degradation.^15^, ^16^ In contrast to NIK, little is known about how the downstream subunit p100/p52 is regulated. βTrCP is required for inducible p100 processing.^17^ Recently, several groups find that FBW7 mediates the complete proteasomal degradation of p100, however, its function in the noncanonical NF‐κB signaling remains controversial.^18^, ^19^, ^20^ Thus, it is important to investigate the molecular mechanism and function of the degradation of p100 in noncanonical NF‐κB activation.

In this study, we identify the E3 ligase TRIM14 as a pivotal positive regulator of noncanonical NF‐κB signaling. The deficiency of TRIM14 down‐regulates noncanonical NF‐κB activation in vitro and in vivo. Mechanistical studies show that TRIM14 recruits USP14 to cleave K63‐linked ubiquitin chains of p100/p52 at lysines 332/338/341, which inhibits the autophagic degradation of p100/p52 by impeding the recognition of the cargo receptor p62. Taken together, our findings provide an insight into the mechanism of intricate regulation of noncanonical NF‐κB signaling through its crosstalk with selective autophagy.

## 2 Results

### 2.1 TRIM14 Potentiates Noncanonical NF‐κB Activation In Vitro and In Vivo

**Figure 1:**
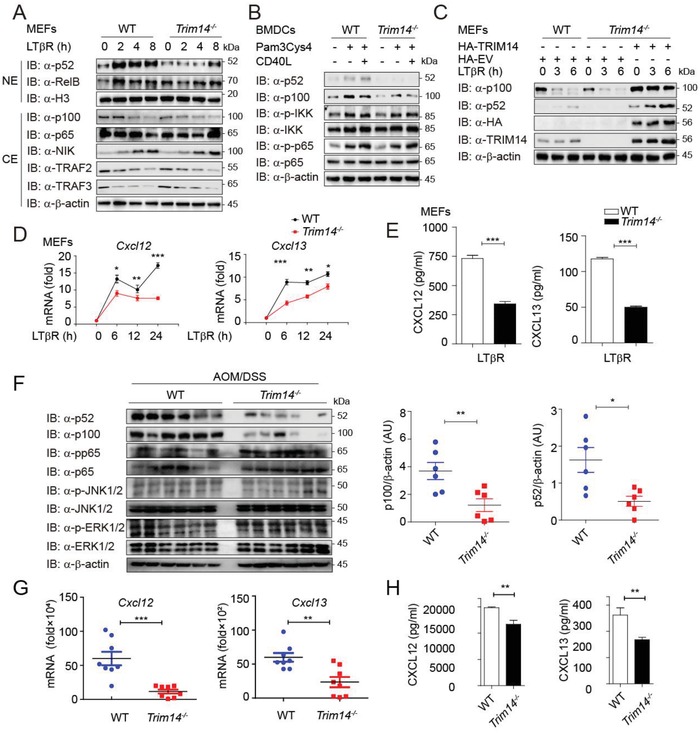
TRIM14 potentiates noncanonical NF‐κB signaling in vitro and in vivo. A) Immunoblot analysis of WT or *Trim14^−/−^* MEFs treated by LTβR for the indicated time periods. B) Immunoblot analysis of WT or *Trim14^−/−^* BMDCs treated by Pam3Cys4 and/or CD40L for 8 h. C) Immunoblot analysis of WT, *Trim14^−/−^* MEFs, or *Trim14^−/−^* MEFs transfected with HA‐TRIM14 or HA‐empty vector (EV), followed by LTβR treatment for the indicated time periods. D) Quantitative polymerase chain reaction (qPCR) analysis of the mRNA level of *Cxcl12*, *Cxcl13* of WT or *Trim14^−/−^* BMDMs or MEFs after TNFα or LTβR stimulation. E) ELISA (enzyme‐linked immunosorbent assay) of cytokine production in WT or *Trim14^−/−^* MEFs treated by LTβR. F) Immunoblot analysis (left) and densitometry (right) of p52, p100, and total β‐actin (loading control throughout) in the distal colon of AOM/DSS‐treated WT and *Trim14^−/−^* mice. AU, arbitrary units. *n* = 6. G) qPCR assays of the mRNA level of *Cxcl12* and *Cxcl13* from colonic tissues. H) ELISA of CXCL12 and CXCL13 in the colon was assessed from tissue culture supernatants. Data in D‐H are presented as means ± SEM of at least three independent experiments. **p < 0.01 and ***p < 0.001, versus the control with the same treatment (Student's *t*‐test).

Previous studies showed that TRIM14 has a critical role for antiviral responses.^21^, ^22^, ^23^ To investigate the function of TRIM14 in NF‐κB signaling and inflammation, we treated wide type (WT) or *Trim14^−/−^* bone‐marrow‐derived macrophages (BMDMs) with lipopolysaccharides (LPS) or TNFα, and found that *Trim14^−/−^* BMDMs displayed only modest differences of the canonical NF‐κB activation, compared with WT BMDMs (Figure S1A,B, Supporting Information). In contrast, compared with *Trim14^−/−^* BMDMs, the protein level of p100 was higher in WT BMDMs (Figure S1A,B, Supporting Information). Since the long‐term stimulation of TNFα could also largely activate noncanonical NF‐κB signaling,^13^, ^24^ we assessed the contribution of TRIM14 in regulating noncanonical NF‐κB pathway by stimulating WT or *Trim14^−/−^* BMDMs with TNFα for longer time periods, and found that TRIM14 depletion decreased the protein level of p100, as well as its protein subunit p52 in response to TNFα (Figure S1C, Supporting Information). TRIM14 had a negligible effect on the activation of mitogen‐activated protein kinases (MAPKs) in response to Toll‐like receptor agonists (Figure S1D, Supporting Information), indicating a specific role for TRIM14 in noncanonical NF‐κB regulation. Most importantly, we found that TRIM14 deficiency in mouse embryonic fibroblasts (MEFs) resulted in a decrease in both the total level of p100 and nuclear p52, but had no effect on the protein level of p65, NIK, TRAF2, or TRAF3 in response to the noncanonical NF‐κB inducer lymphotoxin beta receptor (LTβR; **Figure** **1**A). In addition, TRIM14 deficiency reduced the protein level of both p100 and p52 under CD40L stimulation without affecting the activation of IKK or p65 in the canonical NF‐κB pathway in bone‐marrow‐derived dendritic cells (BMDCs; Figure 1B). Complementation with TRIM14 in *Trim14^−/−^* MEFs restored the protein level of p100 and p52 upon LTβR stimulation (Figure 1C). CXCL12 and CXCL13 are two major chemokines induced by noncanonical NF‐κB signaling.^13^ Indeed, we observed that both the mRNA and protein levels of *Cxcl12* and *Cxcl13* were reduced in TRIM14 deficient cells stimulated with TNFα or LTβR (Figure 1D,E and Figure S1E, Supporting Information). We also evaluated protein level of IL‐6 and TNFα, which are controlled via the canonical NF‐κB pathway.^25^ Levels of IL‐6 and TNFα protein production in WT or TRIM14 deficient cells failed to achieve statistical significance (Figure S1F, Supporting Information).

Since TRIM14 has been studied in different tissues, in which lung, liver, thymus, and spleen display the highest expression,^26^ we next checked whether TRIM14 affects p100 protein level in those tissues. We observed that there was no difference at the mRNA level of *p100* or *Relb* between WT and *Trim14^−/−^* mice (Figure S1G, Supporting Information). Meanwhile, the protein level of p100, but not RelB was down‐regulated in *Trim14^−/−^* mice (Figure S1H, Supporting Information). These results indicated that TRIM14 specifically regulated p100 protein amount in vivo.

A number of reports pointed out that the development of colitis‐associated colon disease models is related to multiple factors from both canonical NF‐κB and noncanonical NF‐κB pathway.^13^, ^27^ To evaluate the role of TRIM14 in NF‐κB signaling in vivo, we applied the inflammation‐driven colitis‐associated cancer (CAC) model^28^ by intraperitoneal subjecting mice with an azoxymethane (AOM) and disuccinimidyl suberate (DSS) treatment together, which could stimulate the colon tumorigenesis (Figure S2A, Supporting Information). AOM and DSS treatment resulted in obvious weight loss in WT mice but not *Trim14^−/−^* mice or control mice from mock group after the initial and third round of DSS treatment (Figure S2B, Supporting Information). Consistent with this result, colons harvested from *Trim14^−/−^* mice were much longer than that from WT mice (Figure S2C, Supporting Information). To assess tumorigenesis, we found that the number and maximal cross‐sectional area of macroscopic polyps were distinctly reduced in *Trim14^−/−^* mice compared to WT mice (Figure S2D, Supporting Information). In addition, we evaluated the expression of key molecules in colon samples harvested from CAC model and found that the ratio of pp65 to p65, pERK1/2 to ERK1/2, or pJNK1/2 to JNK1/2 had no significant difference between *Trim14^−/−^* and WT mice (Figure 1F and Figure S2E, Supporting Information). Without affecting canonical NF‐κB signaling and MAPK signaling, TRIM14 deficiency in mice led to a dramatical reduction in both the protein level of p100 and the production of p52, which has confirmed our hypothesis that TRIM14 mainly played a role in noncanonical NF‐κB signaling by regulating p100/p52. We also found a robust reduction in mRNA expression and protein secretion of CXCL12 and CXCL13 in *Trim14^−/−^* colons (Figure 1G,H). To further confirm whether the function of TRIM14 in colon disease is through the decrease of p100/p52 protein level, we performed DSS‐induced acute colitis in *WT*, *Trim14^−/−^*, and *NFκb2*‐knockdown (KD) chimera mice. *NFκb2*‐KD mice was constructed by bone marrow transplant with *NFκb2* KD‐bone marrow via CRISPR/Cas9 technology, and showed comparable protein level of p100 as in *Trim14^−/−^* chimera mice (Figure S2F,G, Supporting Information). Remarkably, when compared with WT mice, both *Trim14^−/−^* chimera mice and *NFκb2*‐KD chimera mice were considerably more resistant to DSS‐induced acute colitis in both overall survival, weight loss, and colon length (Figure S2H–J, Supporting Information). According to a semi‐quantitative scoring system, *Trim14^−/−^* and *Nfκb2*‐KD chimera mice were presented with decreased clinical severity compared to WT mice (Figure S2K, Supporting Information). Collectively, these data indicate that TRIM14 specifically promotes noncanonical NF‐κB pathway by regulating p100 and p52 protein level in vivo.

### 2.2 TRIM14 Stabilizes p100/p52 through Interaction

**Figure 2:**
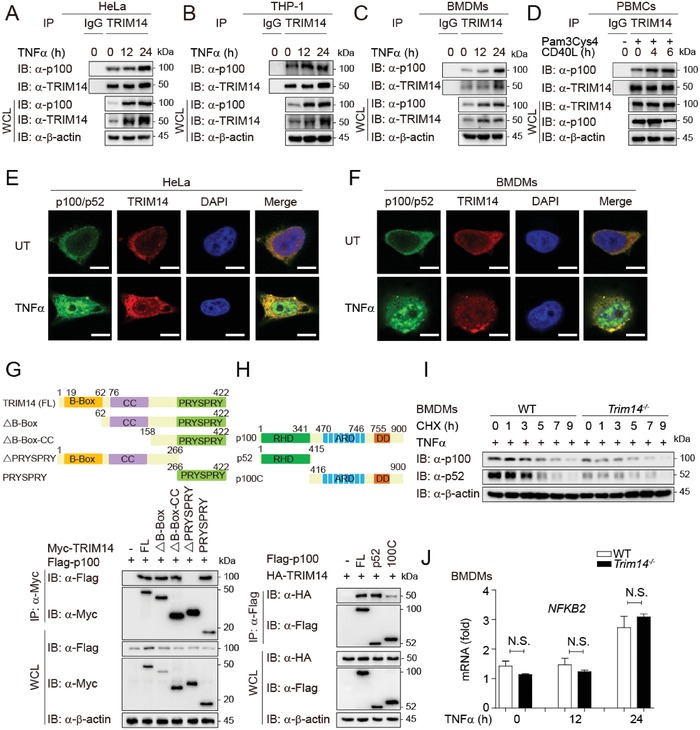
TRIM14 stabilizes p100/p52 through a physical interaction. A–D) Coimmunoprecipitation and immunoblot analysis of extracts of A) HeLa cells, B) THP‐1 cells, C) BMDMs, or D) PBMCs treated with different stimuli for the indicated time periods. WCL, whole‐cell lysates. E,F) Confocal microscopy of E) HeLa cells or F) BMDMs treated with TNFα. Scale bar, 10 µm. G) Coimmunoprecipitation and immunoblot analysis of extracts of HEK293T cells transfected with Flag‐p100 together with Myc‐TRIM14 (FL), Myc‐TRIM14 (∆B‐Box), Myc‐TRIM14 (∆B‐Box‐CC), Myc‐TRIM14 (∆PRYSPRY), or Myc‐TRIM14 (PRYSPRY). H) Coimmunoprecipitation and immunoblot analysis of extracts of HEK293T cells transfected with HA‐TRIM14 together with Flag‐p100 (FL), Flag‐p52 (1–415 aa), Flag‐p100C (416–900 aa). I) Immunoblot analysis of extracts of WT or *Trim14^−/−^* BMDMs treated with TNFα for 24 h, followed with cycloheximide (CHX) for the indicated time periods. J) qPCR analysis of the mRNA level of *Nfκb2* of WT or *Trim14^−/−^* BMDMs after TNFα stimulation. Data in (J) are presented as means ± SEM of at least three independent experiments. N.S., no significant difference.

To investigate the molecular mechanisms by which TRIM14 potentiates noncanonical NF‐κB activation, we performed a coimmunoprecipitation (IP) assay and found that TRIM14 specifically interacted with p52 or p100 rather than other components in noncanonical NF‐κB pathway (Figure S3A, Supporting Information). Moreover, we found that the endogenous p100 interacted with TRIM14 in HeLa, THP‐1, BMDMs, and human peripheral blood mononuclear cells (PBMCs; **Figure** **2**A–D). Confocal microscopy revealed that TRIM14 co‐localized with p100/p52 in the cytoplasm (Figure 2E,F and Figure S3B, Supporting Information) despite that the majority of p52 were translocated into nucleus after TNFα stimulation (Figure S3B, Supporting Information). To identify the domain(s) of p100 or TRIM14 responsible for this interaction, we generated TRIM14 or p100 truncation mutants. The interaction between p100 and TRIM14 was depended on the PRYSPRY domain of TRIM14 (Figure 2G). As expected, the N‐terminal region of p100, which becomes p52 after processing, specifically interacted with TRIM14 (Figure 2H). We next sought to figure out how TRIM14 regulated p100 through this interaction. Previously, we observed that TRIM14 expression increased protein levels of p100/p52 in different types of cells (Figure 1), suggesting a possible role for protein stabilization. Indeed, TRIM14 overexpression inhibited p52 and p100 degradation by a cycloheximide‐chase (CHX) assay (Figure S3C,D, Supporting Information), while TRIM14 deficiency accelerated p52 and p100 degradation (Figure 2I). In contrast, there was no significant difference of *Nfκb2* mRNA level in WT or *Trim14^−/−^* BMDMs (Figure 2J). These data suggest that TRIM14 stabilizes p100/p52 protein level through interaction.

### 2.3 TRIM14 Inhibits Autophagic Degradation of p100/p52

**Figure 3:**
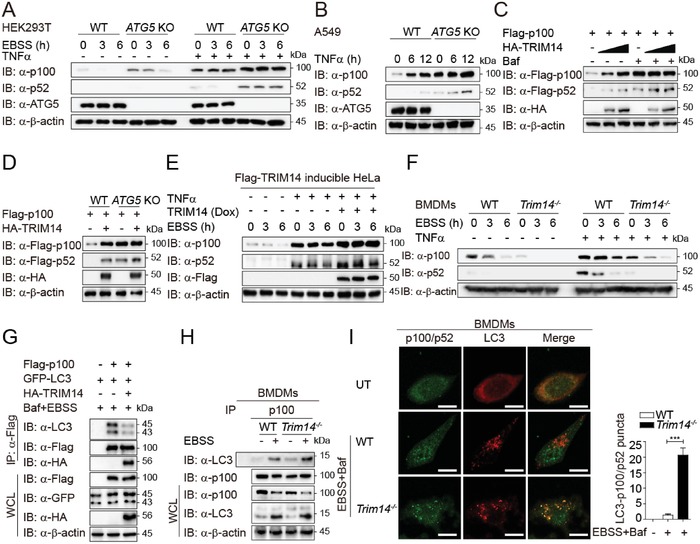
TRIM14 inhibits autophagic degradation of p100/p52. A) Immunoblot analysis of extracts of WT or *ATG5* KO HEK293T cells treated with EBSS along with or without TNFα for the indicated time periods. B) Immunoblot analysis of extracts of WT or *ATG5* KO A549 cells treated with TNFα for the indicated time periods. C) Immunoblot analysis of extracts of HEK293T cells transfected with Flag‐p100, HA‐TRIM14 (wedge) and treated with or without Baf for 8 h. D) Immunoblot analysis of extracts of WT and *ATG5* KO HEK293T cells transfected with Flag‐p100 and HA‐TRIM14. E) Immunoblot analysis of extracts of TRIM14‐inducible HeLa cells treated with or without Dox for 24 h, along with TNFα for 12 h and EBSS for the indicated time periods. F) Immunoblot analysis of extracts of WT or *Trim14^−/−^* BMDMs treated with or without TNFα along with EBSS for the indicated time periods. G) Coimmunoprecipitation and Immunoblot analysis of extracts of HEK293T cells transfected with various combinations of plasmids for Flag‐p100, GFP‐LC3, and HA‐TRIM14 along with EBSS and Baf treatment. H) Coimmunoprecipitation and immunoblot analysis of extracts of WT or *Trim14^−/−^* BMDM cells treated with or without EBSS. I) Confocal microscopy of WT or *Trim14^−/−^* BMDMs treated with or without EBSS or Baf. Scale bar, 10 µm. UT, untreated. Statistics shown refer to the puncta formation by LC3‐p100/p52 in the indicated samples. Data in (I) are presented as means ± SEM of at least three independent experiments (for each treatment, 20 cells in total). ***p < 0.001.

We next investigated how TRIM14 stabilized p100/p52. Three major systems that eukaryotic cells used for protein clearance are the ubiquitin‐proteasome, lysosome, and autolysosome.^29^ We used pharmacologic inhibitors to distinguish which of these systems are responsible for the degradation of p100/p52. p100 could be stabilized with the treatment of the proteasome inhibitor MG132 (Figure S4A, Supporting Information), consistent with previous work.^18^, ^19^ Interestingly, we found both p52 and p100 could also be stabilized by the autophagy inhibitor 3‐methyladenine (3‐MA) or autolysosome inhibitors, chloroquine (CQ) and bafilomycin A1 (Baf; Figure S4A,B, Supporting Information). These data indicate that p100/p52 could be degraded through both proteasome and autophagy pathways. To directly test whether the degradation of p100/p52 occurs via autophagy, we monitored p100/p52 levels in autophagy deficient cells. Strikingly, we found that p100/p52 turnover rates were markedly reduced in *ATG5* knockout (KO) cells upon TNFα stimulation (**Figure** **3**A,B).

We next examined the role of TRIM14 in p100/p52 protein turnover. TRIM14 was not able to further stabilize p100/p52 with the treatment of Baf (Figure 3C), suggesting that TRIM14 specifically inhibits the autophagic degradation of p100/p52. Indeed, TRIM14 failed to stabilize p100/p52 in *ATG5* KO cells (Figure 3D). We treated TRIM14‐inducible cells with doxycycline (Dox) and found that TRIM14 inhibited degradation of p100/p52 induced by amino acid starvation (Figure 3E). Moreover, autophagy‐dependent reduction of p100/p52 was enhanced in *Trim14^−/−^* BMDMs, compared to that in WT BMDMs (Figure 3F). LC3 is a marker protein for autophagy.^30^ We found that TRIM14 expression blocked the interaction of p100 with LC3 in both exogenous and endogenous systems (Figure 3G,H). The association of p52 and LC3 was also attenuated by TRIM14 (Figure S4C, Supporting Information). TRIM14 deficiency enhanced the co‐localization of p100‐LC3 but did not affected global LC3 puncta formation (Figure 3I), consistent with our previous observations.^21^ Taken together, these results indicate that TRIM14 suppresses the selective autophagic degradation of p100/p52.

### 2.4 TRIM14 Impairs p62‐Mediated Selective Autophagic Degradation of p100/p52

**Figure 4:**
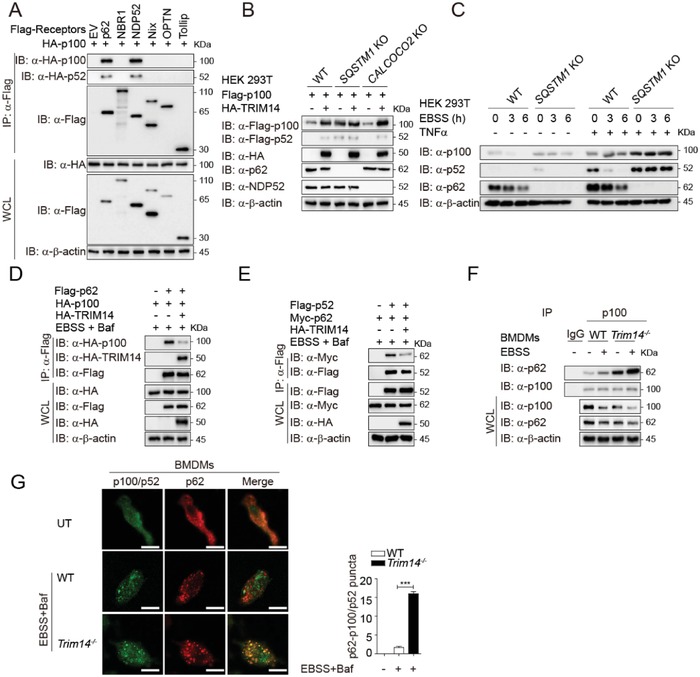
TRIM14 impairs p62‐mediated selective autophagic degradation of p100/p52. A) Coimmunoprecipitation and immunoblot analysis of extracts of HEK293T cells transfected with Flag‐tagged p62, NBR1, NDP52, Nix, OPTN, or Tollip, together with HA‐p100. B) Immunoblot analysis of extracts of WT or *SQSTM1*, *CALCOCO2* KO HEK293T cells transfected with Flag‐p100 and HA‐TRIM14. C) Immunoblot analysis of extracts of WT or *SQSTM1* KO HEK293T cells treated with TNFα or not, followed by EBSS for the indicated time periods. D,E) Coimmunoprecipitation and Immunoblot analysis of extracts of HEK293T cells transfected with D) Flag‐p100 or E) Flag‐p52 along with HA‐TRIM14, Flag‐/Myc‐p62, then treated with Baf and EBSS. F) Immunoprecipitation and immunoblot analysis of extracts of WT and *Trim14^−/−^* BMDMs treated with or without EBSS. G) Confocal microscopy of WT and *Trim14^−/−^* BMDMs treated with Baf and EBSS. Statistics shown refer to the puncta formation by p62‐p100/p52 in the indicated samples. Scale bar, 10 µm. Data in (G) are presented as means ± SEM of at least three independent experiments. ***p < 0.001.

Selective autophagy requires specific cargo receptors that bind both the cargo material and the autophagosomal membrane.^31^ Therefore, we screened the known cargo receptors to identify which were required for p100/p52 degradation. Both p62 (encoded by SQSTM1) and NDP52 (encoded by CALCOCO2) could bind to p100/p52 (**Figure** **4**A and Figure S5A, Supporting Information). However, enhanced p100 and p52 protein levels were detected in *SQSTM1* KO cells but not *CALCOCO2* KO cells (Figure 4B), suggesting that p62 likely recognizes p100/p52 for autophagic degradation. Indeed, p62 deficiency inhibited Earle's balanced salt solution (EBSS)‐mediated degradation of p100/p52 (Figure 4C). Furthermore, the physical association of p100/p52 and p62 was increased during EBSS‐induced autophagy (Figure S5B, Supporting Information). Remarkably, overexpression of TRIM14 impaired the EBSS‐mediated interaction between p100/p52 and p62 (Figure 4D,E), while TRIM14 deficiency enhanced the interaction between p100 and p62 in BMDMs (Figure 4F). Confocal microscopy also showed that p62 and p100/p52 had a stronger co‐localization in TRIM14 deficient cells (Figure 4G). These results suggest that TRIM14 inhibits autophagic degradation of p52/p100 by disturbing the association between p62 and p100/p52.

### 2.5 The K63‐Linked Ubiquitination of p52/p100 at Lysines 332/338/341 Is a Signal for Autophagic Degradation

**Figure 5:**
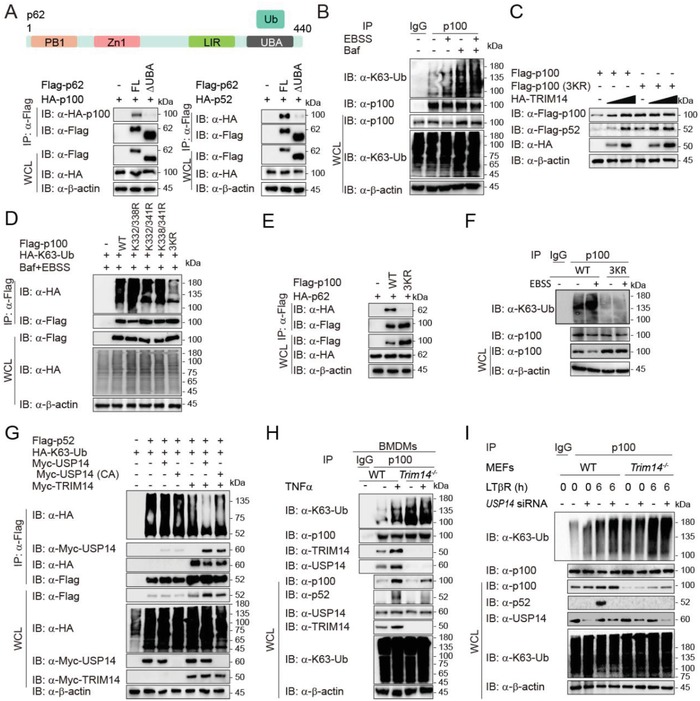
TRIM14 inhibits K63‐linked ubiquitination of p100/p52 at K332/338/341 by recruiting USP14. A) Coimmunoprecipitation and immunoblot analysis of extracts of HEK293T cells transfected with various combinations of plasmids for HA‐p52 or p100 and Flag‐p62 constructs. B) Coimmunoprecipitation and immunoblot analysis of extracts of MEFs treated with Baf or EBSS. C) Immunoblot analysis of extracts of HEK293T cells transfected with various combinations of plasmids for HA‐TRIM14 (wedge), Flag‐p100, and Flag‐p100 (3KR, K332/338/341R). D) Coimmunoprecipitation and immunoblot analysis of extracts of HEK293T cells transfected with Flag‐p100, the indicated p100 mutants, HA‐K63‐ubiquitin (Ub), then treated with Baf and EBSS. E) Coimmunoprecipitation and immunoblot analysis of extracts of HEK293T cells transfected with Flag‐p100 (3KR) or Flag‐p100 (WT) along with HA‐p62. F) Coimmunoprecipitation and immunoblot analysis of extracts of p100‐WT and p100‐3KR knock‐in THP‐1 cells with or without EBSS treatment. G) Coimmunoprecipitation and immunoblot analysis of HEK293T cells transfected with various combinations of plasmids for Flag‐p52, HA‐K63‐Ub, Myc‐USP14, Myc‐USP14 (CA), and Myc‐TRIM14. CA, C114A. H) Coimmunoprecipitation and immunoblot analysis of extracts of WT or *Trim14^−/−^* BMDMs treated with TNFα or not. I) Coimmunoprecipitation and immunoblot analysis of extracts of WT or *Trim14^−/−^* MEFs transfected with *USP14* siRNA and followed with LTβR treatment for 6 h.

The cargo receptor p62 recognizes ubiquitinated protein targets through its UBA (Ub‐associated) domain.^32^ We found that deletion of p62 UBA domain abrogated the interaction between p62 and p100/p52 (**Figure** **5**A), suggesting that the ubiquitination of p100/p52 is a pivotal signal for p62‐dependent selective autophagy. Indeed, starvation‐induced autophagy dramatically enhanced the ubiquitination of p100/p52, and ubiquitinated p100/p52 accumulated in the presence of the autophagy inhibitor Baf (Figure S6A, Supporting Information), indicating that ubiquitinated p100/p52 undergoes robust autophagic degradation. We next found that starvation‐induced autophagy enhanced K63‐linked ubiquitination of p100, but not other types of ubiquitin chains (Figure 5B and Figure S6B, Supporting Information). This is consistent with a report that oligomeric p62 preferentially binds to mono‐ubiquitin or K63‐linked ubiquitin chains rather than K48‐linked ubiquitin chains during selective autophagy.^33^

To define the relevant ubiquitination site, we identified 17 conservative lysine sites of p100 in silico (Figure S6C, Supporting Information) and generated the corresponding lysine (K)‐to‐arginine (R) substitution in every possible site. Although initial results showed that TRIM14 could stabilize all the p100 mutants, we noticed that protein levels of p100 K332R, K338R, or K341R mutants were higher than that of WT p100 (Figure S6D, Supporting Information), suggesting that the regulation of p100 through TRIM14 might not be dependent on single‐site ubiquitination. Therefore, we generated double and triple lysine mutants of p100 (Figure S6E, Supporting Information) and found that TRIM14 could no longer stabilize the mutant containing arginine substitutions at K332, K338, and K341 (3KR; Figure 5C and Figure S6E, Supporting Information). Moreover, K63‐linked ubiquitination of the p100 3KR mutant was much lower than WT p100 (Figure 5D). Consistently, the p100 3KR mutant was not able to interact with p62 (Figure 5E). To further confirm the role of p100/p52, we then constructed a p100‐3KR knock‐in THP1 cell line. Compared to p100 in WT cells, p100‐3KR was more stabilized and easily processed into p52 by TNFα treatment (Figure S6F, Supporting Information), and the K63‐linked ubiquitination level of p100‐3KR was much lower than it from WT p100, which was consistent with the previous results (Figure 5F). Furthermore, the expression and secretion of Cxcl12 and Cxcl13 were significantly higher in the 3KR cells, compared to that in WT cells (Figure S6G‐H, Supporting Information). Collectively, these results indicate that K63‐linked ubiquitin chains at K332/338/341 of p100/p52 are essential for the recognition and degradation of p100/p52 by p62‐dependent selective autophagy.

### 2.6 TRIM14 Inhibits the K63‐Linked Ubiquitinationof p100/p52 by Recruiting USP14

We next examined whether TRIM14 stabilizes p100/p52 by inhibiting the K63‐linked ubiquitination of p100/p52. Since TRIM14 is not a deubiquitinating enzyme (DUB),^22^ we speculated that TRIM14 might recruit certain DUBs to deubiquitinate p100/p52. According to the previous study,^21^ USP14 and BRCC3 are two major TRIM14‐interacting DUBs. Here, we found that USP14 rather than BRCC3 enhanced p100 and p52 protein levels in the presence of TRIM14, and that was dependent upon its catalytic activity (Figure S7A, Supporting Information). USP14 interacted with full length (FL) p100 and p52, but not the C‐terminal region of p100, and this interaction was augmented by TRIM14 (Figure S7B, Supporting Information). The interaction between USP14 and p52 facilitated TRIM14‐mediated de‐ubiquitination and stabilization of p52, which also required the protease activity of USP14 (Figure 5G). In addition, USP14 did not affect the ubiquitination of p100 3KR mutant, even in the presence of TRIM14 (Figure S7C, Supporting Information), indicating that USP14 specifically cleaves the ubiquitin chains on K332/338/341 of p100/p52. We also confirmed that TRIM14 depletion not only disrupted the association between USP14 and p100/p52, but also enhanced p100 ubiquitination, and thus leading to the degradation of p100/p52 (Figure 5H). In the absence of USP14, TRIM14 failed to inhibit K63‐linked ubiquitination of p100 and stabilize p100/p52 (Figure S7D, Supporting Information), while USP14 alone could not deubiquitinate p100 and stabilize p100/p52 without TRIM14 (Figure 5I).

We next observed that USP14 deficiency results in the decreased level of p100/p52, but not other noncanonical NF‐κB regulators, including NIK or RelB in MEFs (Figure S8A, Supporting Information). Moreover, we observed that the reduced expression of USP14 attenuated both the mRNA level and protein level of Cxcl12 and Cxcl13 in MEFs (Figure S8B,C, Supporting Information). These results further support the function of TRIM14‐USP14 complex in noncanonical NF‐κB pathway, by showing that USP14 is also indispensable in regulating p100 or p52 stabilization in noncanonical NF‐κB pathway. Taken together, these results indicate that TRIM14 recruits USP14 to cleave K63‐linked ubiquitin chains of p100/p52 at K332/338/341 and inhibit the p62‐dependent autophagic degradation of p100/p52.

## 3 Conclusion

**Figure 6:**
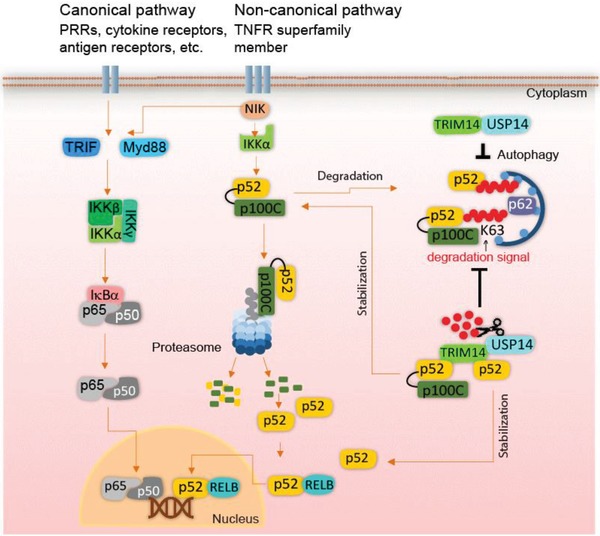
Schematic representation of noncanonical NF‐κB signaling positively regulated by TRIM14‐USP14 axis.

NF‐κB pathways have multiple roles in physiological and pathological processes, including cell proliferation, immune response, and inflammation.^1^, ^3^ The tight regulation of NF‐κB largely depends on the post‐translational modifications (PTMs) of signaling components which form a complicated network of protein turnover.^34^ Unlike canonical NF‐κB signaling which has been extensively investigated, the regulation of noncanonical NF‐κB pathway has just started to be appreciated in recent years.^3^, ^35^ Currently, most studies concerning noncanonical NF‐κB regulation focus on the regulation of NIK, which is destabilized by TRAF3‐mediated proteasomal degradation under normal conditions.^12^ However, little is known about the regulation of the actual transcriptional regulators of noncanonical NF‐κB signaling. We identified TRIM14 as a novel positive regulator of noncanonical NF‐κB signaling by regulating the stability of transcriptional regulators p100/p52. The genetic ablation of murine TRIM14 dramatically inhibits the activation of noncanonical NF‐κB pathway in vivo. Mechanistically, we found that TRIM14 interacts with p100/p52 through its PRYSPRY domain. TRIM14 inhibits the p62‐mediated autophagic degradation of p100/p52 by recruiting USP14 to cleave K63‐linked ubiquitin chains at K332/338/341 of p100/p52. The stabilization of p100/p52 by TRIM14‐USP14 complex subsequently enhances noncanonical NF‐κB signaling (**Figure** **6**). Thus, the TRIM14‐USP14 complex promotes the activation of noncanonical NF‐κB pathway through the crosstalk with autophagy.

Autophagy is an essential homeostatic process for clearance of intracellular protein aggregates, injured organelles, and invading cytoplasmic microbes.^36^ Recently, increasing evidence suggests the important regulatory roles of the crosstalk between autophagy and a variety of cellular responses, including immune responses and inflammation.^37^ For example, selective autophagy plays a pivotal role in regulating type I interferon signaling^21^, ^38^ and canonical NF‐κB signaling^39^ as described by our previous studies. Here, our findings suggest that p62‐mediated autophagic degradation of p100/p52 negatively regulates the noncanonical NF‐κB signaling, and this process is specifically inhibited by TRIM14. Our study provides new insights into the crosstalk between autophagy and noncanonical NF‐κB signaling.

It has been reported that FBXW7α (also known as FBW7) targets nuclear p100 for proteasomal degradation, which depends on phosphorylation of p100 by GSK3.^18^ In our study, we found that TRIM14‐USP14 axis inhibits the degradation of p100/p52 to promote noncanonical NF‐κB signaling. Intriguingly, it has been reported that Akt not only inhibits the function of GSK3 but also enhances the deubiquitinase activity of USP14 by phosphorylation.^40^ We speculated that the protein level of p100/p52 regulated by TRIM14/USP14 or GSK/ FBXW7α might be tightly controlled by Akt pathway, which helps to maintain homeostasis of the intracellular signaling. The involvement of AKT in the crosstalk between selective autophagy and noncanonical NF‐κB signaling might require further investigation.

Ubiquitination has emerged as an irreplaceable PTM in a variety of cellular processes, which is tightly regulated by a complex network. Various regulators of ubiquitination have cooperated together to modulate intracellular responses. Our previous data have also shown that TRIM14 recruits USP14 to accelerate the stabilization of cGAS in response to virus infection,^21^ as a pair of mutual binding partners, TRIM14‐USP14 in this context has been convinced to regulate noncanonical NF‐κB signaling through the control of selective autophagy. TRIM14 is an inducible gene by both type I interferon^21^, ^22^ and noncanonical NF‐κB signaling activation (Figure 2A,B), which may be important to turn on the downstream signal by bridging USP14 and the target protein cGAS or p100 in the cytoplasm. Indeed, TRIM14‐USP14 complex is not a unique model in the regulation of innate immune signaling. For example, TRIM27‐USP7 complex inhibited the function of TBK1 in type I interferon signaling by enhancing the ubiquitination of TBK1.^41^ In another study, the ubiquitination–deubiquitination cascade mediated by TRIM27‐USP7 complex plays an essential role in TNFα‐induced apoptosis.^42^ Collectively, we speculate that TRIM14‐USP14 complex is capable of targeting different proteins in innate immunity. The unique role of TRIM14 prompts us to explore novel therapies for diseases related to innate immune responses and autophagy.

## 4 Experimental Section

*Reagents*: Expression plasmids were transfected with StarFect high‐efficiency transfection reagent (GenStar) according to the manufacturer's instruction. To induce starvation, cells were washed with phosphate‐buffered saline (PBS, Gibco) and incubated in EBSS (Gibco). Puromycin (P9620, 1–5 µg mL^−1^), MG132 (C‐2211‐5MG, 10 × 10^−6^ m), Dox (D9891, 100 ng mL^−1^), Baf (H2714, 0.2 × 10^−6^ m), chloroquine phosphate (CQ, PHR1258‐1G, 50 × 10^−6^ m) and 3‐MA (M9281‐100MG, 10 × 10^−6^ m), LPS (L4391‐1MG, 100 ng mL^−1^), cycloheximide from microbial (CHX, C1988‐1 g, 100 µg mL^−1^), AOM (A5486‐25MG) were purchased from Sigma. Pam3CSK4 (Pam3Cys4, 112208‐00‐1, 25 ng mL^−1^) was purchased from Invivogen. Recombinant TNFα (300‐01A, 10 ng mL^−1^) and recombinant murine CD40 ligand (315‐15, 500 ng mL^−1^) were purchased from PeproTech; anti‐LTBR (ab65089, 25 µg mL^−1^) was purchased from Abcam; DSS (21655) was purchased from Thermo fisher.

*Mice and Animal Experiments*: C57BL/6 *Trim14* ^−/−^ mice were generated by CRISPR/Cas9 system as described previously.^21^ *Nfκb2*‐KD mice were constructed by bone marrow transplant (WT bone marrow transfected with CRIPSR‐Cas9‐sg*Nfκb2*, guide RNA: 5′‐CGTTGTACTCGCGAGCTAGGGGG‐3′). All the experimental procedures involving animal studies were performed according to the National Act on the use of experimental animals (China). These procedures were approved by the Laboratory Animal Center of Sun Yat‐Sen University.

*Isolation of PBMCs, BMDMs, BMDCs, and MEFs*: Blood from healthy donors (Zhongshan School of Medicine) was used for the isolation of peripheral blood mononuclear cells (PBMCs) by ficoll‐hypaque density‐gradient centrifugation. The use of PBMCs was in compliance with institutional guidelines and approved protocols by Sun Yat‐Sen University. All healthy donors signed a consent form approved by the Research Ethics Committee of the Sun Yat‐sen University Cancer Center (GZR2013‐040). The isolation of BMDMs, BMDCs, and MEFs was performed as described previously.^21^, ^41^ To complement TRIM14 in *Trim14^−/−^* MEFs, TRIM14 was transfected with jetPRIME (polyplus‐transfection) according to the manufacturer's protocols. Cells were collected at 24–30 h after transfection.

*Induction of CAC in Mice*: For CAC model, mice were i.p. injected with mutagen AOM (10 mg kg^−1^ body weight, Sigma Aldrich), then followed with three cycles of 2% DSS treatment. Mice on regular drinking water were used as vehicle controls.

*Colon Organ Culture*: Colons from three models were assessed, washed several times in cold PBS containing 10% EBSS (Sigma‐Aldrich), and cultured for 24 h in RPMI media (Gibco) containing 2 × penicillin‐streptomycin (Gibco) at 37 °C. Supernatants were collected for cytokines detection.

*Macroscopic Polyp Analysis*: In the CAC model, mice were euthanized and the entire colon was removed on Day 58, after washed several times, colons were opened longitudinally for assessing macroscopic polyp formation.^13^

*Induction of DSS‐Induced Acute Colitis in Mice*: To induce acute colitis, mice were administrated 2.5% DSS (mol. wt. 35 000–50 000 Da; MP Biomedicals) in their drinking water ad libitum for 5 days and euthanized on Day 10.

*Statistical Analysis*: The results of all quantitative experiments were reported as mean ± SEM of three independent experiments, and Student's *t*‐test was used for all statistical analyses with the GraphPad Prism 5.0 software.

## Conflict of Interest

The authors declare no conflict of interest.

## Author Contributions

M.C., Z.Z., Q.M., and P.L. contributed equally to this work. M.C., Z.Z., Q.M., and P.L. performed the experiments and analyzed the results. Z.S. and J.H. provided the technical help. J.C. initiated and designed the project and directed the research. M.C., Z.Z., and J.C. wrote the manuscript.

## Supporting information

Supporting Information
