# Supplementary material for: TRIM14 Promotes Noncanonical NF‐κB Activation by Modulating p100/p52 Stability via Selective Autophagy
Source: Adv Sci (Weinh). 2019 Nov 11;7(1):1901261. doi: 10.1002/advs.201901261 (PMC6947505; doi:10.1002/advs.201901261)
Supplement: Supplementary file 1 — Supporting Information [file ADVS-7-1901261-s001.pdf]

Copyright WILEY-VCH Verlag GmbH & Co. KGaA, 69469 Weinheim, Germany, 2019.

ADVANCED SCIENCE
Open Access

Supporting Information

for *Adv. Sci.*, DOI: 10.1002/advs.201901261

TRIM14 Promotes Non-canonical NF-κB Activation by Modulating p100/p52 stability via Selective Autophagy

Meixin Chen[1,3], *Zhiyao Zhao*[1,2,3], *Qingcai Meng*[1,3], *Puping Liang*[1,3], *Zexiong Su*[1], *Yaoxing Wu*[1], *Junjiu Huang*[1], and *Jun Cui*[1*]

Copyright WILEY-VCH Verlag GmbH & Co. KGaA, 69469 Weinheim, Germany, 2019.

Supporting Information

TRIM14 Promotes Non-canonical NF-κB Activation by Modulating p100/p52 stability via Selective Autophagy

Meixin Chen[1,3], *Zhiyao Zhao*[1,2,3], *Qingcai Meng*[1,3], *Puping Liang*[1,3], *Zexiong Su*[1], *Yaoxing Wu*[1], *Junjiu Huang*[1], and *Jun Cui*[1*]

[1] State Key Laboratory of Oncology in South China, MOE Key Laboratory of Gene Function and Regulation, School of Life Sciences, Sun Yat-sen University, Guangzhou, Guangdong, 510006, China. [2] Department of Internal Medicine, Guangzhou Institute of Pediatrics, Guangzhou Women and Children's Medical Center [3] These authors contributed equally to this work.

Contents of supporting information

Supporting Figure 1-8

Extended experimental procedures

Figure S1

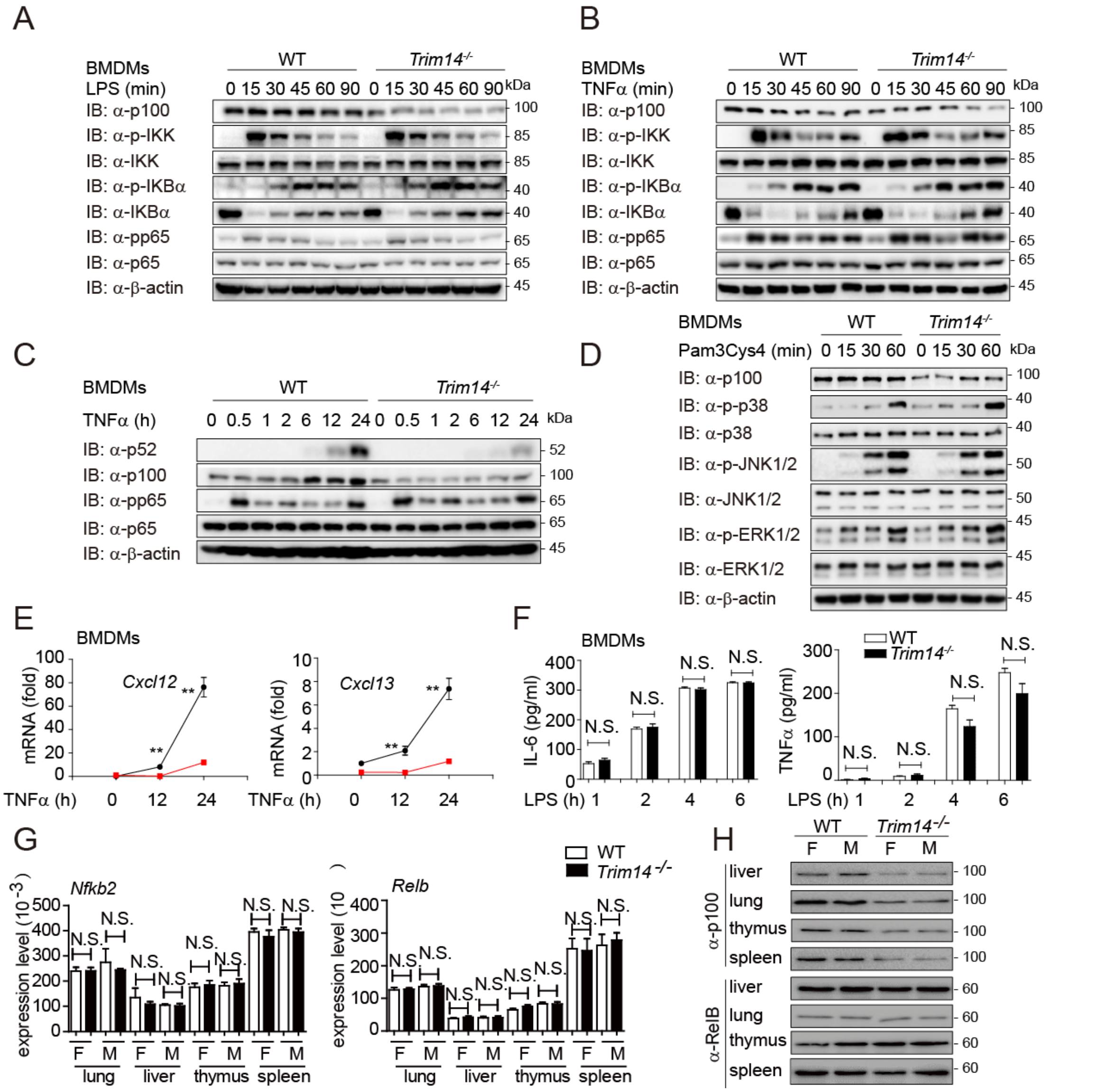

Figure S1, TRIM14 deficiency specifically affects non-canonical NF-κB signaling via down-regulating the protein amount of p100/p52. (A, B) Immunoblot analysis of wild type (WT) or *Trim14*$^{-/-}$ BMDMs treated by LPS (A) or TNFα (B) for the indicated time periods. **(C)** Immunoblot analysis of wild type (WT) or *Trim14*$^{-/-}$ BMDMs treated by TNFα for the indicated time periods. **(D)** Immunoblot analysis of WT or *Trim14*$^{-/-}$ BMDMs treated by Pam3Cys4 for the indicated time periods. **(E)** qPCR analysis of the mRNA level of *Cxcl12*, *Cxcl13* of WT or *Trim14*$^{-/-}$ BMDMs or MEFs after TNFα stimulation. **(F)** ELISA of IL-6, TNFα production of WT or *Trim14*$^{-/-}$ BMDMs

after LPS stimulation. **(G)** qPCR analysis of the mRNA level of *RelB* and *Nfkb2* of indicated tissues from WT or *Trim14*$^{-/-}$ mice. **(H)** Immunoblot analysis of extracts of indicated tissues from WT or *Trim14*$^{-/-}$ mice (each sample was mixed tissue from three mice). F, female, M, male. Data in E-H is presented as the means ± SEM of three independent experiments. *p < 0.05, **p < 0.01, ***p < 0.001, N.S., no significant difference, versus the control cells with the same treatment (Student's t-test).

Figure S2

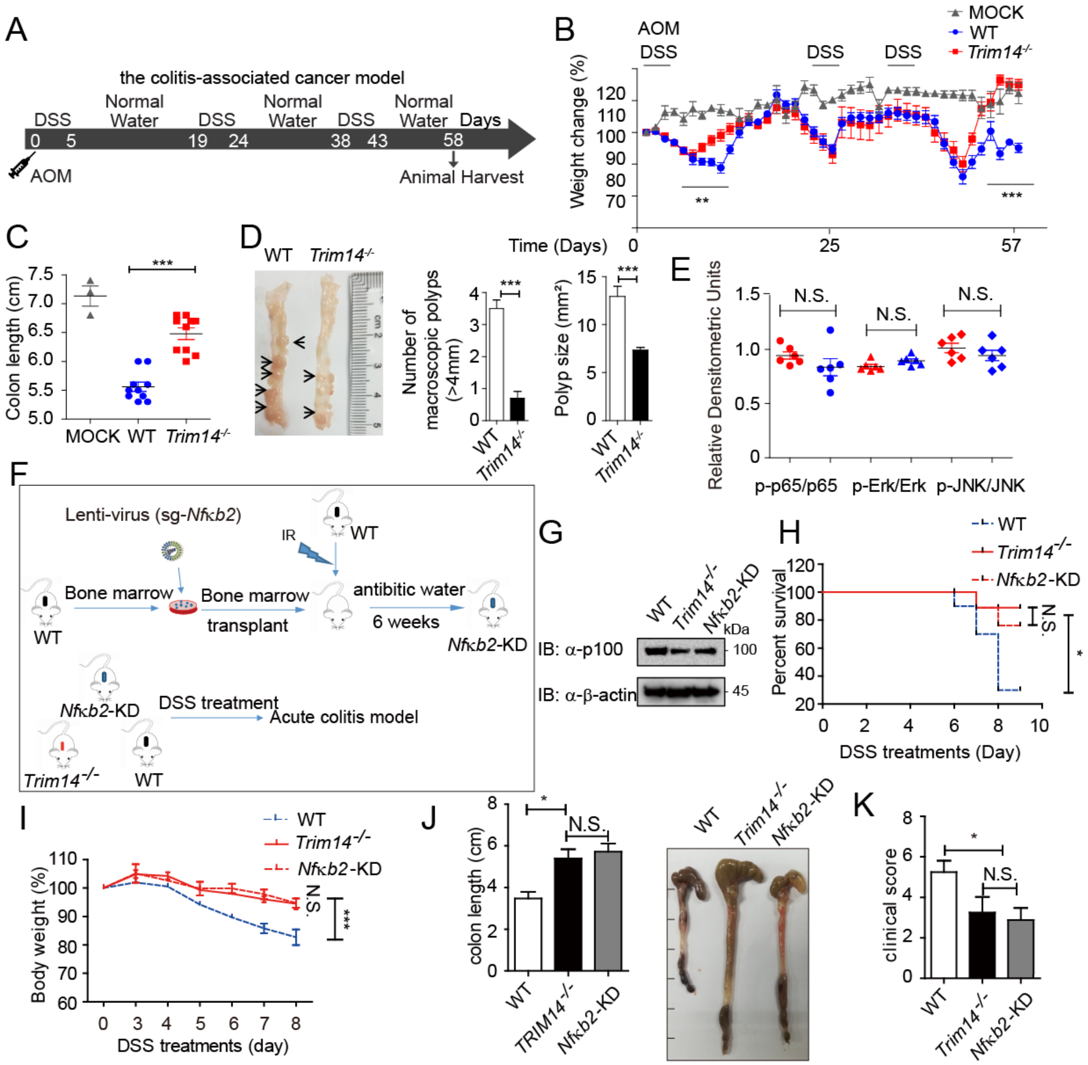

Figure S2, TRIM14 enhances symptoms in the colitis-associated cancer model. (A) Schematic of the colitis-associated cancer model. **(B)** Weight loss was monitored throughout the AOM/DSS model. **(C)** Colon length from AOM/DSS-treated *Trim14*$^{-/-}$ and WT mice. **(D)** Macroscopic polyps (arrows) were identified in the distal and middle colons, harvested from *Trim14*$^{-/-}$ and WT mice. The number and maximal cross-sectional area of macroscopic polyps was quantified. **(E)** The protein levels of pp65, pERK1/2 and pJNK1/2 in the distal colon of AOM/DSS-treated WT and *Trim14*$^{-/-}$ mice in Figure 1F were evaluated. **(F)** Schematic of *NFκB2*-KD mice generation and DSS-induced acute colitis. WT, *Trim14*$^{-/-}$ and *NFκB2*-KD mice were challenged with 2.5% dextran sulfate sodium (DSS) for 5 days and disease progression was assessed daily. *Trim14*$^{-/-}$, n=6; WT,

n=6; *NFκB2*-KD, n=6. **(G)** Protein level of p100 in bone marrow from indicated mice. **(H-K)** Survival **(H)**, weight loss **(I)**, colon length **(J)** and clinical scores **(K)** in WT, *Trim14*$^{-/-}$ and *NFκB2*-KD mice from the acute colitis model. Data in **B**-**E**, **H**-**K** are presented as means ± SD of the experiments with 8 or 6 mice per group. *p < 0.05, **p < 0.01, ***p < 0.001, N.S., no significant difference, versus the wild type or control group with the same treatment (student's t-test in **B**-**E**, **I**-**K** and Mantel-Cox test and Gehan-Breslow-Wilcoxon test in **H**).

Figure S3

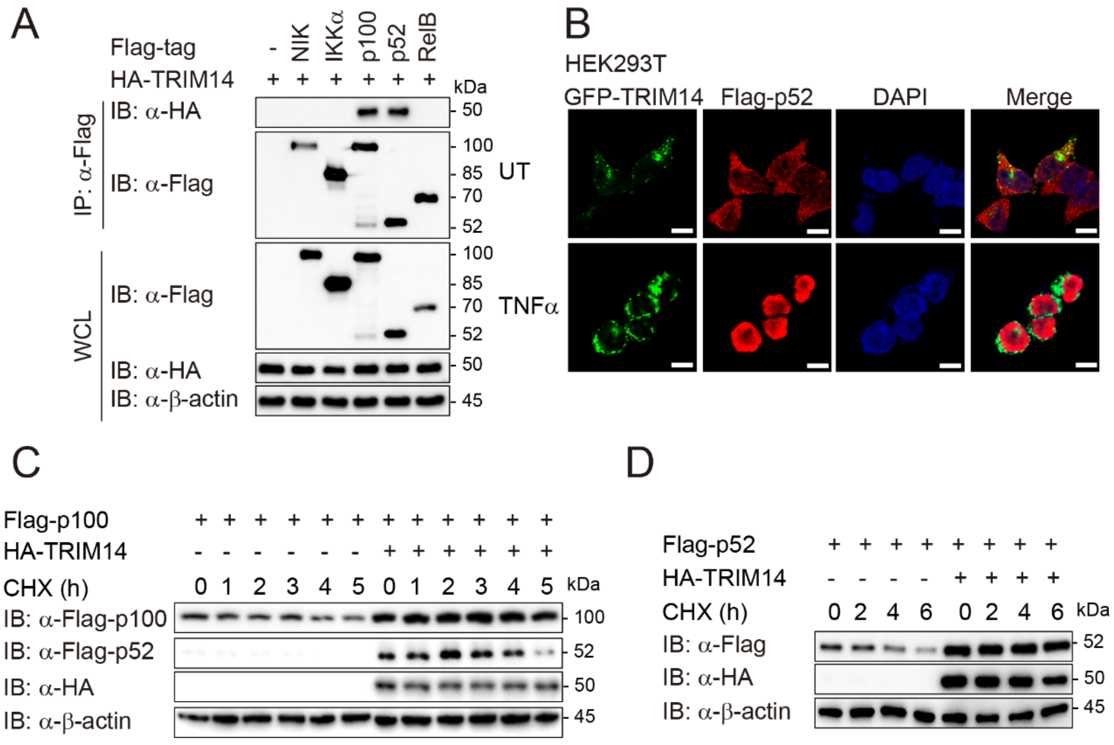

Figure S3, TRIM14 stabilizes p100/p52 through interaction. (A) Coimmunoprecipitation and Immunoblot analysis of extracts of HEK293T cells transfected with HA-TRIM14 together with Flag-tagged NIK, IKKα, p100, p52 and RelB. **(B)** Confocal microscopy of HEK293T cells transfected with Flag-p52, GFP-TRIM14, followed by TNFα treatment. Scale bar, 10 μm. **(C, D)** Immunoblot analysis of extracts of HEK293T cells transfected with Flag-p100 (C) or Flag-p52 (D) along HA-TRIM14, then treated with cycloheximide (CHX) for the indicated time periods.

Figure S4

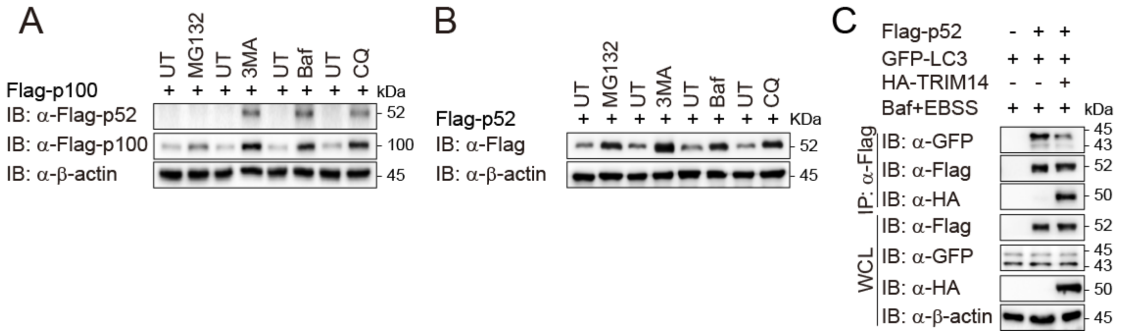

Figure S4, TRIM14 inhibits autophagy-mediated but not proteasome-dependent degradation of p100/p52. (A-B) Immunoblot analysis of extracts of HEK293T cells transfected with Flag-p100 (A) or Flag-p52 (B) and treated with or without MG132, 3-methyladenine (3-MA), bafilomycin A1 (Baf) or chloroquine phosphate (CQ). **(C)** Coimmunoprecipitation and Immunoblot analysis of extracts of HEK293T cells transfected with Flag-p52, HA-TRIM14, GFP-LC3, and treated with Baf and EBSS.

Figure S5

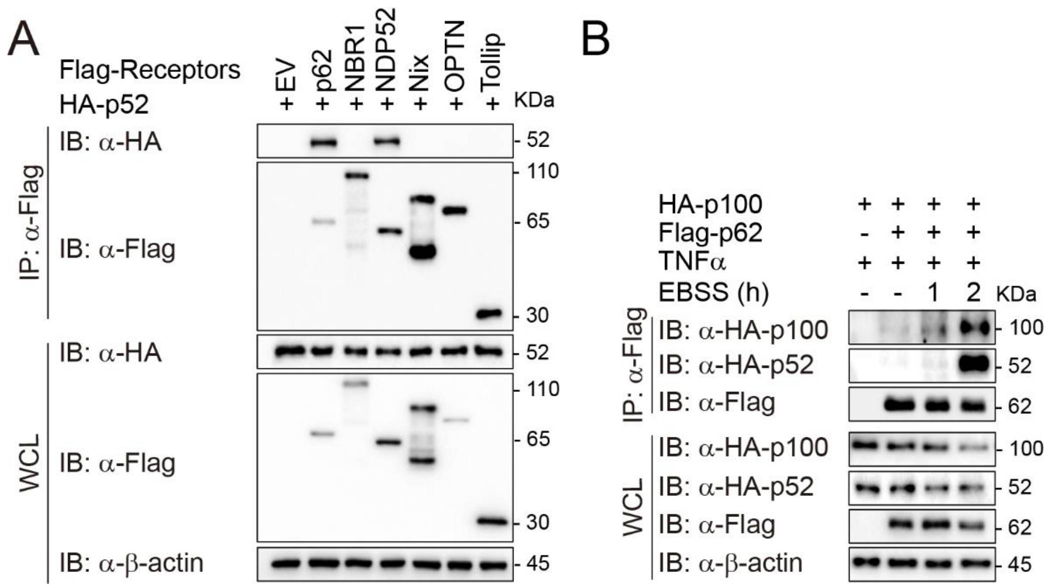

Figure S5, p100/p52 degraded through p62-mediated autophagy. (A) Coimmunoprecipitation and Immunoblot analysis of extracts of HEK293T cells transfected with Flag-tagged p62, NBR1, NDP52, Nix, OPTN, or Tollip together with HA-p52. **(B)** Immunoprecipitation and Immunoblot analysis of extracts of HEK293T cells transfected with HA-p100, Flag-p62, and treated with TNFα or EBSS for 0, 1, or 2 h.

Figure S6

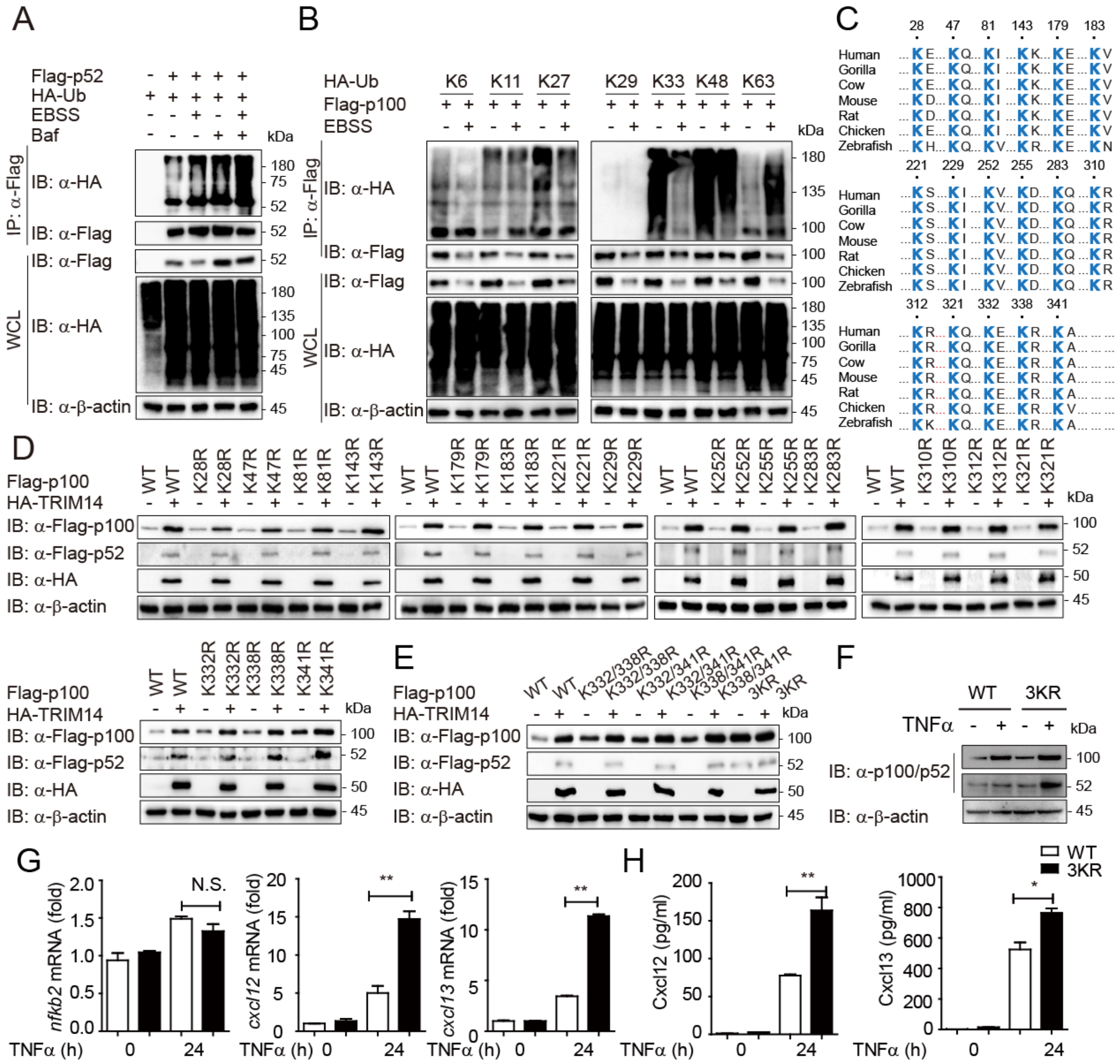

Figure S6, TRIM14 inhibits K63-Linked ubiquitination of p100/p52 at K332/338/341. (A) Coimmunoprecipitation and Immunoblot analysis of extracts of HEK293T cells transfected with Flag-p52, HA-ubiquitin (Ub), treated with EBSS or Baf. **(B)** Coimmunoprecipitation and Immunoblot analysis of extracts of HEK293T cells transfected with Flag-p100 and HA-tagged WT Ub or ubiquitin mutants (K6, K11, K27, K29, K33, K48, K63), then treated with EBSS. **(C)** Alignment of p52 amino acid sequences. **(D, E)** Immunoblot analysis of extracts of HEK 293T cells transfected with HA-TRIM14, Flag-p100 and the indicated mutants. **(F)** Immunoblot analysis of extracts

of *NFKB2* knockout THP1 cells constituted with WT, p100-3KR (K332/338/341R) and followed by TNFα treatments. **(G)** qPCR analysis of the mRNA level of *nfkb2*, *cxcl12* and *cxcl13* from the same sample of (F). **(H)** ELISA of Cxcl12 and Cxcl13 in supernatants from (F). Data in G-H is presented as the means ± SEM of three independent experiments. *$p < 0.05$, **$p < 0.01$, ***$p < 0.001$, N.S., no significant difference, versus the control cells with the same treatment (Student's t-test).

Figure S7

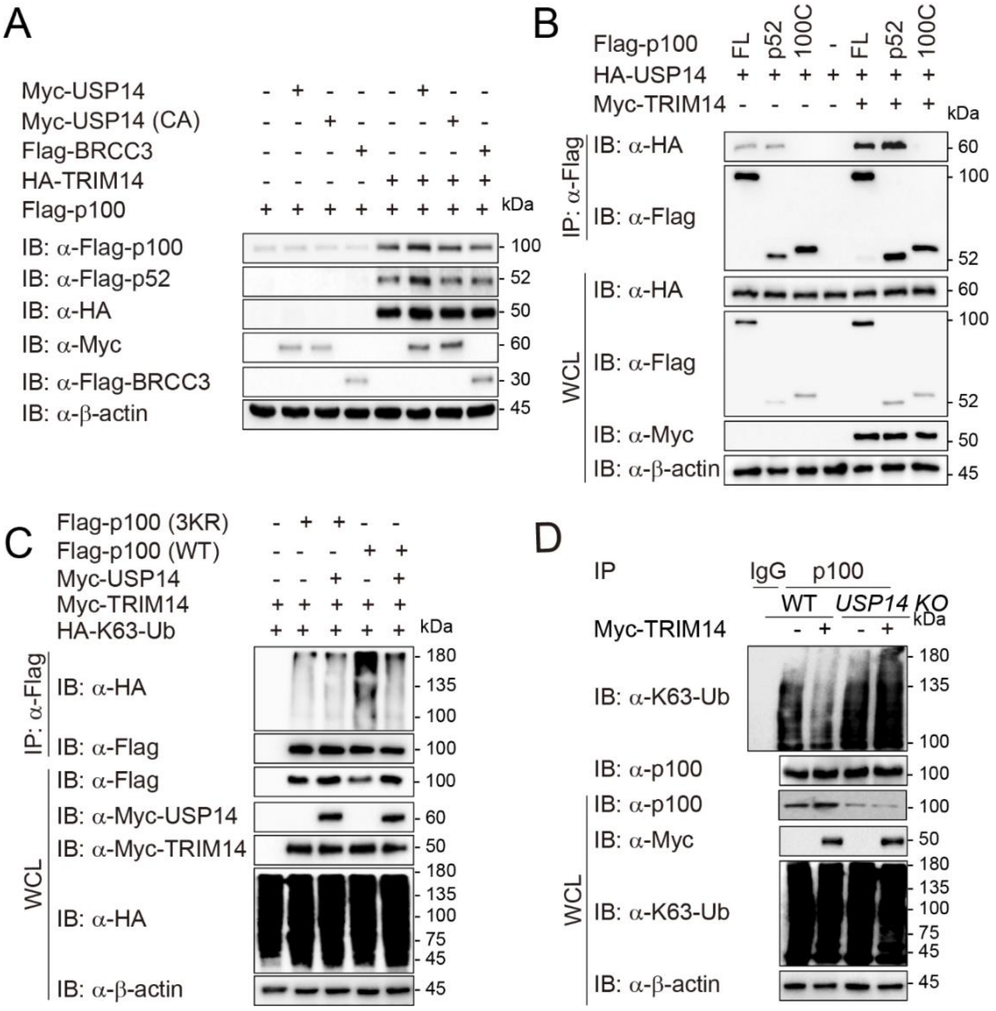

Figure S7, TRIM14 inhibits K63-Linked ubiquitination of p100/p52 by recruiting USP14. (A) Immunoblot analysis of extracts of HEK293T cells transfected with various combinations of plasmids for Flag-p100, HA-TRIM14, Myc-USP14, Myc-USP14 (CA) and Flag-BRCC3. CA, C114A. **(B)** Coimmunoprecipitation and immunoblot analysis of HEK293T cells transfected with various combinations of plasmids for Flag-p100, Flag-p52, Flag-p100C, HA-USP14 and Myc-TRIM14. **(C)** Coimmunoprecipitation and immunoblot analysis of extracts of HEK293T cells transfected with various combinations of plasmids for Flag-p100 (WT), Flag-p100 (3KR, K332/338/341R), HA-K63-Ub along with Myc-USP14 and Myc-TRIM14. **(D)** Coimmunoprecipitation and immunoblot analysis of extracts of WT and *USP14* KO HEK293T cells.

Figure S8

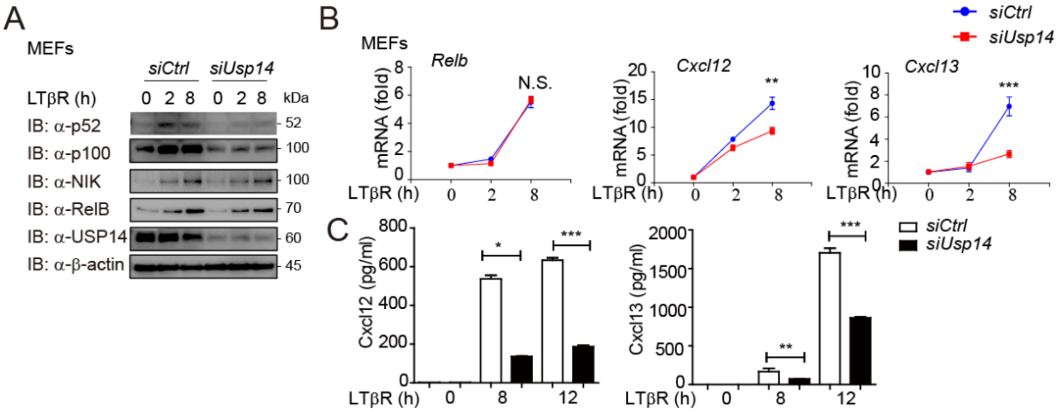

Figure S8, USP14 deficiency impairs non-canonical NF-κB signaling. (A) Immunoblot analysis of extracts of MEFs transfected with control siRNA (*siCtrl*) or *Usp14*-specific siRNA (*siUSP14*) and followed by LTβR treatments. **(B)** qPCR analysis of the mRNA level of *cxcl12* and *cxcl13* from the cells in (A). **(C)** ELISA of cytokine production in the supernatants from MEFs transfected with *siCtrl* or *siUSP14* and followed by LTβR treatments. Data in B-C is presented as the means ± SEM of three independent experiments. *$p < 0.05$, **$p < 0.01$, ***$p < 0.001$, N.S., no significant difference, versus the control cells with the same treatment (Student's t-test).

EXTENDED EXPERIMENTAL PROCEDURES

Cell culture

HEK 293T, HeLa, A549, THP-1 cells, human peripheral blood mononuclear cells (PBMCs), bone marrow derived macrophages (BMDMs), and bone-marrow derived dendritic cells (BMDCs) were cultivated in Dulbecco's modified Eagle's medium (HyClone) or RPMI-1640 medium (Gibco) supplemented with 10% fetal bovine serum (GenStar) and 1% L-glutamine (Gibco) at 37°C in 5% CO_2.

Reagents

Expression plasmids were transfected with StarFect High-efficiency Transfection Reagent (GenStar) according to the manufacturer's instruction. To induce starvation, cells were washed with phosphate-buffered saline (PBS) (Gibco) and incubated in Earle's balanced salt solution (EBSS) (Gibco). Puromycin (P9620), MG132 (C-2211-5MG), doxycycline (Dox; D9891), bafilomycin A1 (H2714), chloroquine phosphate (CQ) (PHR1258-1G) and 3-methyladenine (3-MA) (M9281-100MG), lipopolysaccharides (LPS, L4391-1MG), cycloheximide from microbial (CHX, C1988-1g), azoxymethane (AOM, A5486-25MG) were purchased from Sigma. Pam3CSK4 (Pam3Cys4, 112208-00-1) was purchased from Invivogen. Recombinant TNF-α (300–01A) and recombinant murine sCD40 ligand (315-15) were purchased from PeproTech; Anti-LTBR (ab65089) was purchased from Abcam; Disuccinimidyl suberate (DSS, 21655) was purchased from Thermo fisher.

Antibodies

The goat polyclonal antibodies against p52/p100 (NFκB p52 Antibody (C-5) (sc-7386) and NFKB2 Polyclonal Antibody (A5567-50u) were from Santa Cruz and Abclonal respectively; anti-TRIM14 (sc-79761), anti-Ubiquitin (sc-8017), anti-RelB (sc-226X), donkey anti-goat IgG-HRP, goat anti-mouse IgG-HRP, goat anti-rabbit IgG-HRP were from Santa Cruz; Anti-K63-linkage poly-ubiquitin (D7A11) from Cell Signaling Technology; Anti-phospho-IKKα/β (2697S), anti-phospho-JNK (9251), anti-JNK (9252), anti-phospho-ERK (9101), anti-ERK (9102), anti-phospho-p38 (9211), anti-p38 (9212), anti-p65 (6956), anti-phospho-p65(3031), anti-IκBα (4814) and anti-phospho-IκBα

(9246) were from Cell Signaling Technology; anti-TRAF2 (A0962), anti-TRAF3 (A3094) from Abclonal; Anti-ATG5 (12994S), anti-Beclin 1 (3738), anti-LC3 (PM036) and anti-p62 (8025) were purchased from Cell Signaling Technology; USP14 (WH0009097M4-100UG), horseradish peroxidase (HRP)-anti-Flag (M2) (A8592) and anti-β-actin (A1978) were purchased from Sigma; HRP-anti-hemagglutinin (12013819001), anti-c-Myc-HRP (11814150001) were purchased from Roche Applied Science; Anti-NDP52 (12229-1-AP) was purchased from Proteintech Group. Goat and Rabbit IgG were from Beyotime. Protein G agarose and Protein A agarose were purchased from Pierce. The secondary antibodies for immunofluorescence were purchased from Biotium. Anti-IKKα (IMG-136A) WAS from IMGENEX. Anti-IKKβ (05-535) WAS from MILLIPORE. TriMethyl-Histone H3-K4 Polyclonal Antibody (A2357) was ABclonal.

Plasmids

p100, TRIM14 and other plasmids mentioned were acquired by the means of standard PCR techniques. Point mutations of some plasmids were generated by site-directed mutagenesis (Sbsgene), the primers used are as follows:

p100 (K28R) forward: CCATTGTGGAACCCAGGGAGCCAGCCCCAGAAACAGC

p100 (K28R) reverse: GTTTCTGGGGCTGGCTCCCTGGGTTCCACAATGG

p100 (K47R) forward:

GGTGATCGTGGAACAGCCTAGGCAGAGAGGCTTCCG

p100 (K47R) reverse:

CGAAATCGGAAGCCTCTCTGCCTAGGCTGTTCCACGATC

p100 (K81R) forward: GACCTATCCCACTGTCAGGATCTGTAACTACGAGGG

p100 (K81R) reverse: GGTCCCTCGTAGTTACAGATCCTGACAGTGGGATAGG

p100 (K143R) forward: GTCCTGCATGTGACTAGGAAGAACATGATGGG

p100 (K143R) reverse: GTCCCCATCATGTTCTTCCTAGTCACATGCAGG

p100 (K179R) forward: GCTGGAGCAAGAGGCCAGAGAACTGAAGAAGGTG

p100 (K179R) reverse: CCATCACCTTCTTCAGTTCTCTGGCCTCTTGCTCC

p100 (K183R) forward: GGCCAAAGAACTGAAGAGGGTGATGGATCTG

p100 (K183R) reverse: CTCAGATCCATCACCCTCTTCAGTTCTTTGG

p100 (K221R) forward: GCCCATCCATGACAGCAGATCTCCGGGGGCATC

p100 (K221R) reverse: GGTTTGATGCCCCCGGAGATCTGCTGTCATGGATGG

p100 (K229R) forward: GGGCATCAAACCTGAGGATTTCTCGAATGG

p100 (K229R) reverse: GTCCATTCGAGAAATCCTCAGGTTTGATGC

p100 (K252R) forward: TATCTGCTTTGTGACA**G**GGTGCAGAAAGATGAC

p100 (K252R) reverse: GTCATCTTTCTGCACC**C**TGTCACAAAGCAGATA

p100 (K255R) forward: TGTGACAAGGTGCAGA**G**AGATGACATTGAGGTT

p100 (K255R) reverse: AACCTCAATGTCATCT**C**TCTGCACCTTGTCACA

p100 (K283R) forward: CCCACAGATGTGCATAGACAGTATGCCATTGTG

p100 (K283R) reverse: CACAATGGCATACTGTCTATGCACATCTGTGGG

p100 (K310R) forward: GTGTTTCTGCAACTGAGACGCAAGCGAGGAGGG

p100 (K310R) reverse: CCCTCCTCGCTTGCGT**C**TCAGTTGCAGAAACAC

p100 (K312R) forward:

TTTCTGCAACTGAAACGCAGGCGAGGAGGGGACGTGTCT

p100 (K312R) reverse:

AGACACGTCCCCTCCTCGCCTGCGTTTCAGTTGCAGAAA

p100 (K321R) forward: GACGTGTCTGATTCCAGACAGTTCACCTATTAC

p100 (K321R) reverse: GTAATAGGTGAACTGTCTGGAATCAGACACGTC
p100 (K332R) forward:

TACCCTCTGGTGGAAGACAGGGAAGAGGTGCAGCGGAAG

p100 (K332R) reverse:

CTTCCGCTGCACCTCTTCCCTGTCTTCCACCAGAGGGTA

p100 (K338R) forward: AAGAGGTGCAGCGGAGGCGGAGGAAGGCCTT

p100 (K338R) reverse: AAGGCCTTCCTCCGCCTCCGCTGCACCTCTT
p100 (K341R) forward: CAGCGGAAGCGGAGGAGGGCCTTGCCCACCTTC

p100 (K341R) reverse: GAAGGTGGGCAAGGCCCTCCTCCGCTTCCGCTG

p100 (S707A) forward: CCTGTGCCCACTGCCTGCACCCCCTACCTCTGA

p100 (S707A) reverse: TCAGAGGTAGGGGGTGCAGGCAGTGGGCACAGG

p100 (S711A) forward: GCCTTCACCCCCTACCGCTGATAGCGACTCGGA

p100 (S711A) reverse: TCCGAGTCGCTATCAGCGGTAGGGGGTGAAGGC

Generation of knockout cells by CRISPR/Cas9 technology

USP14, ATG5 knockout cells were generated with a CRISPR/Cas9 system. The sequences of target related gene are as follows:

USP14-guide RNA: *5'*-GGAATGACTCTACTAATGAT-3*'*.

ATG5-guide RNA: *5'*-GTGCTTCGAGATGTGTGGTT-3*'*.

RNA extraction and quantitative RT-PCR

Total cellular RNA was isolated by TRIzol Reagent (Invitrogen), and first-strand cDNA was generated from total RNA using oligo-dT primers and reverse transcriptase (TAKARA). Real-time PCR was performed with the SYBR Green qPCR Mix (GenStar) and specific primers using the Primer5 analyzer (Applied Biosystems). Data were normalized to GAPDH gene, and the relative abundance of transcripts was calculated by the Ct models. The following primers were used for real-time PCR:

mouse CXCL12 forward primer, 5’ GATGATCCCAATGAGTAGGC3’

mouse CXCL12 reverse primer, 5’ TGTATGTCTGGACCCATTCC3’

mouse CXCL13 forward primer, 5’ GGCCACGGTATTCTGGAAGC 3’

mouse CXCL13 reverse primer, 5’ ACCGACAACAGTTGAAATCACTC 3’

mouse NFKB2 forward primer, 5’ CATCCATGACAGCAAGTCTC 3’

mouse NFKB2 reverse primer, 5’ TCCTCATAGAACCGAACCTC 3’

mouse GAPDH forward primer, 5’ GAAGGGCTCATGACCACAGT 3’

mouse GAPDH reverse primer, 5’ GGATGCAGGGATGATGTTCT 3’

Measurement of cytokines

Concentrations of the cytokines in cell culture supernatants or mouse serum were determined by ELISA kits (BD Biosciences or Elabscience), according to the manufacturer’s recommendations.

Immunoprecipitation and immunoblotting

For immunoprecipitation, whole-cell extracts were acquired after transfection or stimulation with appropriate ligands, followed by incubation overnight with anti-Flag

(Sigma) or the appropriate antibodies plus Protein A/G beads (Pierce). Beads were then washed 5 times with low-salt lysis buffer, and immunoprecipitates were eluted with 2×SDS Loading Buffer (Cell Signaling Technology) and resolved by SDS-PAGE. Proteins were transferred to polyvinylidene fluoride membranes (Bio-Rad Laboratories) and further incubated with the appropriate antibodies. LumiGlo Chemiluminescent Substrate System (KPL) was used for protein detection.

Two-step immunoprecipitation and ubiquitination assays

The ubiquitination levels of p100/p52 in this study was examined by two-step immunoprecipitation and ubiquitination assays, which were performed according to the previous report [54]. For the first-step immunoprecipitation assay, whole-cell extracts were prepared by using low-salt buffer supplemented with a 5 mg/ml protease inhibitor cocktail (Roche). Lysates were incubated with the anti-Flag beads overnight. The immunoprecipitates were washed 3 to 5 times with low salt buffer. For the second-step immunoprecipitation assay, the immunoprecipitates were denatured by boiling for 5 minutes in the Lysis buffer containing 1% SDS. The elutes were diluted 1:10 with low-salt buffer. The diluted elutes were re-immunoprecipitated with anti-Flag beads overnight. After 3 to 5 times wash, the immunoprecipitates were resolved by SDS-PAGE.

Immunofluorescence

BMDMs, HeLa cells or HEK293T cells seeded on Glass Bottom culture dishes (Nest Scientific) were fixed with 4% paraformaldehyde for 15 minutes, and then permeabilized in methyl alcohol for 10 minutes at -20℃. After washing with PBS for 3 times, cells were

blocked in 5% fetal goat serum for 1 hour, and then incubated with primary antibodies diluted in 10% bull serum albumin overnight. The cells were washed and followed by a fluorescently labeled secondary antibody (Alexa Fluor 488- and Alexa Fluor 568-conjugated antibodies against mouse, rabbit or goat IgG (Biotium)). Confocal images were examined using a microscope (LSM710; Carl Zeiss) equipped with 100× 1.40 NA oil objectives, with Immersol 518F (Carl Zeiss) as imaging medium and a camera (AxioCam HRc; Carl Zeiss) under the control of Zen 2008 software (Carl Zeiss). The images were processed for gamma adjustments using LSM Zen 2008 or ImageJ software (National Institutes of Health).
